# Temporal Dynamics of ICP, CPP, PRx, and CPPopt in High-Grade Aneurysmal Subarachnoid Hemorrhage and the Relation to Clinical Outcome

**DOI:** 10.1007/s12028-020-01162-4

**Published:** 2021-01-09

**Authors:** Teodor Svedung Wettervik, Timothy Howells, Anders Lewén, Elisabeth Ronne-Engström, Per Enblad

**Affiliations:** Department of Neuroscience, Section of Neurosurgery, Uppsala University, 751 85 Uppsala, Sweden

**Keywords:** Cerebral perfusion pressure, Clinical outcome, Neurointensive care, Pressure autoregulation, Secondary insults, Subarachnoid hemorrhage

## Abstract

**Background:**

High intracranial pressure (ICP) and low cerebral perfusion pressure (CPP) may induce secondary brain injury following aneurysmal subarachnoid hemorrhage (aSAH). In the current study, we aimed to determine the temporal incidence of insults above/below certain ICP/CPP thresholds, the role of pressure autoregulation in CPP management (PRx and CPPopt), and the relation to clinical outcome.

**Methods:**

In this retrospective study, 242 patients were included with aSAH, who were treated in the neurointensive care unit, Uppsala University Hospital, Sweden, 2008–2018, with ICP monitoring the first 10 days post-ictus. Data from ICP, pressure autoregulation (PRx), CPP, and CPPopt (the CPP with the lowest/optimal PRx) were analyzed the first 10 days. The percentage of good monitoring time (GMT) above/below various ICP and CPP thresholds was calculated, e.g., ICP > 20 mm Hg (%), CPP < 60 mm Hg (%), and ∆CPPopt (CPP–CPPopt) < − 10 mm Hg (%).

**Results:**

Of the 242 patients, 63 (26%) had favorable (GOS-E 5–8) and 179 (74%) had unfavorable (GOS-E 1–4) outcome at 12 months. Higher proportion (GMT) of ICP insults above 20 mm Hg was most common the first 3 days post-ictus and was then independently associated with unfavorable outcome. CPP gradually increased throughout the 10 days post-ictus, and higher proportion of GMT with CPP < 90 mm Hg was independently associated with unfavorable outcome in the late vasospasm phase (days 6.5–10). PRx was above 0 throughout the 10 days and deteriorated in the late vasospasm phase. Higher values were then independently associated with unfavorable outcome. There was no difference in GMT of CPP deviations from CPPopt between the outcome groups.

**Conclusions:**

Avoiding intracranial hypertension early and maintaining a high CPP in the vasospasm phase when the pressure autoregulation is most disturbed may improve clinical outcome after aSAH.

## Introduction

Aneurysmal subarachnoid hemorrhage (aSAH) constitutes 5% of all cases of stroke and is associated with high mortality and severe neurological sequelae [1]. Clinical management aims at early aneurysm occlusion, cerebrospinal fluid (CSF) diversion in case of acute hydrocephalus, and avoiding delayed cerebral ischemia (DCI) [2–5]. Treatment in the neurointensive care (NIC) has generally led to better clinical outcome for these patients [3, 4, 6]. In an early NIC study, we found that a higher incidence of secondary insults in general was associated with unfavorable outcome after aSAH [3]. Specifically, secondary intracranial pressure (ICP) insults above 20 and 25 mm Hg are relatively common post-ictus [4, 7–9] and associated with clinical deterioration [4] and mortality [7], but the relation to favorable/unfavorable clinical outcome is less clear [4, 7, 8]. Regarding cerebral perfusion pressure (CPP; mean arterial blood pressure—ICP) insults, the evidence for the benefit of fixed CPP thresholds is low. CPPs above 60–70 mm Hg are generally desired [4, 10] and levels below 70 mm Hg have been suggested to be associated with brain tissue hypoxia and energy metabolic disturbances due to impaired cerebral blood flow (CBF) [10]. However, aSAH has a dynamic CBF pathophysiology with cerebral large-vessel vasospasm that peaks days 4 to 10 post-ictus and that contributes to the development of DCI [5, 11]. The implication of this is that the goal for CPP treatment could vary.

One way of monitoring the changes in the CBF pathophysiology is continuous assessment of the pressure autoregulatory status, which could ultimately lead to a more individualized and dynamic CPP management. In TBI, pressure autoregulation has been monitored using the pressure reactivity index (PRx), i.e., a correlation index between ICP and mean arterial blood pressure (MAP). Negative values, e.g., when a decrease in MAP leads to cerebral vasodilation to maintain CBF with a corresponding increase in cerebral blood volume and ICP, indicate preserved pressure autoregulation and are strongly associated with favorable outcome in TBI [12, 13]. As PRx varies with CPP in a U-shaped way, the CPP with the concurrently lowest PRx/best autoregulation has been suggested as optimal (CPPopt). Deviation of absolute CPP from CPPopt has been associated with poor clinical outcome in TBI [13–15]. Some studies suggest that disturbed PRx values and deviation from CPPopt targets are associated with low CBF in aSAH [16, 17], but their ability to predict DCI and clinical outcome are less clear [16–22].

Although ICP/CPP insults and disturbances in cerebral autoregulation are common following aSAH, their temporal evolution and relation to clinical outcome are not fully elucidated. In the current study, our aim was to evaluate the temporal incidence of ICP and CPP insults based on different threshold values and the role of pressure autoregulation in relation to clinical outcome. We were particularly interested in evaluating fixed CPP thresholds as compared to the autoregulatory CPPopt thresholds in relation to clinical outcome.

## Materials and Methods

### Patients

Patients with aSAH admitted to the Department of Neurosurgery at the University Hospital in Uppsala, Sweden, 2008–2018 were eligible for this study. Out of 605 patients with SAH and ICP monitoring, we included 242 aSAH patients aged 16 or older with ICP/CPP monitoring on all of the first 10 days post-ictus.

### Treatment Protocol

Patients were treated in accordance with our standardized ICP- and CPP-oriented treatment protocol to avoid secondary insults [4]. The treatment protocol remained unchanged throughout the study period. Treatment goals were ICP ≤ 20 mm Hg, CPP ≥ 60 mm Hg, systolic blood pressure > 100 mm Hg, central venous pressure (CVP) 0–5 mm Hg before the aneurysm was occluded and 5–10 mm Hg afterward, pO_2_ > 12 kPa, arterial glucose 5–10 mmol/L (mM), electrolytes within normal ranges, normovolemia and body temperature < 38 °C.

Patients who were unconscious (GCS M < 6) were intubated and mechanically normoventilated. Those patients were sedated with propofol and received morphine as analgesia. Wake-up tests were repeatedly performed. The patients were treated with early aneurysm occlusion, including endovascular embolization or surgical clipping, and all patients received nimodipine (first as infusion of 2 mg/h and later as tablets 60 mg × 6 for 3 weeks in total). The dosage of infusion was reduced temporarily in case of hypotension to avoid negative hemodynamic effects. In unconscious (GCS M < 6) patients, an external ventricular drain (EVD) was inserted to monitor and to drain cerebrospinal fluid (CSF) in case of high ICP. The EVD was initially kept closed to measure ICP and assess the need for CSF drainage. If ICP was above 20 mm Hg the EVD was opened at 15 mm Hg. In severe cases when basal ICP treatment was insufficient, thiopental coma treatment and/or decompressive craniectomy (DC) were last-tier treatments. Arterial blood pressure was maintained with fluids. Inotropes (dobutamine or in second hand norepinephrine) were only used if CPP was below 60 mm Hg and the patient did not respond to intravenous fluid treatment.

DCI was defined as new neurological deficits and/or decreased level of consciousness when other causes, e.g., hydrocephalus and hematomas, were excluded. If a manifest cerebral infarction was excluded, triple-H therapy (hypertension, hypervolemia, and hemodilution) including 500 ml dextran-40 solution (100 mg/ml, Meda AB) and 200 ml albumin (200 mg/ml) were administered for 5 days. Cerebral intra-arterial nimodipine was given in case of refractory vasospasm and angioplasty was performed in case of refractory large-vessel vasospasm. The main target for triple-H therapy was elevation of blood pressure but only to moderately elevated levels in relation to baseline, i.e., in general CPP to around 70–80 mm Hg and systolic blood pressure to around 140–160 mm Hg. Secondary targets were erythrocyte volume fraction (EVF) 32% and CVP 8–14 mm Hg, although these goals were in general met automatically by the fluid therapy given. The targeted levels were increased stepwise if clinical improvement was not seen.

### Data Acquisition and Analyses

The data were collected using our software Odin, developed at Edinburgh and Uppsala University by one of the authors (TH) [23]. ICP was monitored with the EVD system (HanniSet, Xtrans, Smith Medical GmbH, Grasbrunn, Germany). Arterial blood pressure was measured invasively in the radial artery at heart level. The sampling frequency was 100 Hz. Pressure reactivity index (PRx) was calculated as the 5-min correlation of 10 s averages of ICP and MAP [12, 13]. CPPopt was calculated continuously, minute-by-minute as the CPP with the lowest PRx the last 4 h, as described by Aries et al. [14]. Daily CPPopt-values could be calculated in 90–99% of the patients, depending on the day. However, CPPopt could only be calculated for 54% of the GMT for the entire cohort the first 10 days. The data acquisition was on some occasions interrupted when the patients left the NIC (e.g., for surgery) or due to technical aspects (e.g., network failure). These interruptions and data that were judged invalid were subtracted from the total monitoring time, resulting in the good monitoring time (GMT).

### Outcome

Clinical outcome was assessed at around 12 months post-hemorrhage, by specially trained personnel using structured telephone interviews for the Extended Glasgow Outcome Scale (GOS-E) containing eight categories of outcome, from death to upper good recovery [24, 25]. GOS-E 5–8 was considered favorable clinical outcome, whereas GOS-E 1–4 was considered unfavorable.

### Statistical Analysis

The temporal dynamics of the following physiological variables were studied in relation to clinical outcome after aSAH: MAP, ICP, CPP, CPPopt, PRx, and body temperature.

Nominal, ordinal, and continuous variables were described as numbers or proportions, medians (interquartile range (IQR)) and means (± standard deviation), respectively. Mean daily values for MAP, ICP, CPP, CPPopt, PRx, and body temperature were evaluated day-to-day the first 10 days post-ictus for those with favorable/unfavorable outcome and those with triple-H/no triple-H with 95% confidence interval (CI).

The 10-day period post-ictus was divided into three phases—1. Early phase (days 1 to 3), 2. Early vasospasm phase (days 4 to 6.5), and 3. Late vasospasm phase (days 6.5 to 10). The vasospasm phase was hence split in the middle at 6.5 days. The proportion (%) of GMT with secondary insults of ICP, CPP, and body temperature above/below certain predefined thresholds were calculated for each phase. We defined ICP insults as ICP above threshold values at 20 mm Hg and 25 mm Hg, respectively. The former was chosen in accordance with our management protocol and the latter as a “severe” insult. We studied GMT (%) of CPP below threshold values at 60 mm Hg, 70 mm Hg, 80 mm Hg, and 90 mm Hg. We chose 60 mm Hg in accordance with our management protocol and then increased the threshold with 10 mm Hg up to 90 mm Hg. We defined autoregulatory CPP thresholds as optimal when the absolute CPP was within ± 10 mm Hg from CPPopt (∆CPPopt = CPP–CPPopt ± 10 mm Hg), whereas ∆CPPopt < -10 mm Hg and ∆CPPopt > 10 mm Hg were considered as hypo- and hyperperfusion insults. We defined hyperthermia insults as body temperature above 38 °C in accordance with our management protocol. There is no gold standard PRx threshold of autoregulation. We therefore focused on mean values, but evaluated also PRx > 0.05 which has been used to dichotomize TBI patients in favorable/unfavorable outcome [26]. We compared the incidence of ICP, CPP, hyperthermia, and PRx insults and in each of three phases for those with favorable and unfavorable outcome. Those secondary insults that were significant in the univariate analyses in each phase were analyzed in a multiple logistic regression in addition to age, World federation of neurosurgical society (WFNS) grade, and Fisher grade as explanatory variables for favorable outcome. If, e.g., both ICP thresholds (20 and 25 mm Hg) were significant in the univariate analysis in one phase, each threshold was analyzed in the multiple regression separately and the regression with the highest area under receiver operating curve (AUROC) was chosen as optimal. A *p *value < 0.05 was considered statistically significant. As this was an exploratory study, we did not adjust for multiple comparisons.

### Ethics

All procedures performed in the studies involving humans were in accordance with the ethical standards of the national research committee and with the 1964 Helsinki declaration and its later amendments. The study was approved by Uppsala University Regional Ethical Board (Dnr 2010/138 and Dnr 2010/138/1). Informed consent was obtained during neurointensive care from the next of kin.

## Results

### Demography, Admission Status and Treatments, and Relation to Clinical Outcome

There were 242 patients included in the study (Table 1). Mean age was 58 (± 11) and the female/male ratio was 163/79 (67/33%). Median GCS M was 5 (IQR 5–6) and pupillary abnormalities (anisocoria/fixed) were present in 13 (5%) patients at admission. There were 183 (76%) patients with a WFNS grade above III and 110 (55%) patients with a Hunt and Hess grade above III. Median Fisher grade was 4 (IQR 3–4) and the aneurysm was located in the anterior cerebral circulation for 196 (81%) patients and in the posterior circulation for the remaining patients. The majority (n = 169 (70%)) of the patients were treated with endovascular aneurysm occlusion, whereas 67 (28%) were treated with clip ligation, 2 (1%) patients with both methods, and 4 (2%) patients received no aneurysm occlusion. All patients were intubated and they were mechanically ventilated in median 12 (IQR 9–15) days. Sixty-one (25%) patients were treated with triple-H therapy due to DCI, of whom 6 also received cerebral intra-arterial nimodipine and 1 received additional angioplasty. Twenty-four (10%) patients were treated with thiopental and 34 (14%) with DC. Ten (4%) patients were treated with both thiopental and DC.

**Table 1:** Demography, admission status, and treatments—relation to clinical outcome

	All	Favorable	Unfavorable	*p* Value
Patients, *n* (%)	242 (100%)	63 (26%)	179 (74%)	NA
Age, mean (± SD) years	58 (± 11)	53 (± 11)	60 (± 10)	***0.001***
Sex (female/male), *n* (%)	163/79 (67/33%)	43/20 (68/32%)	120/59 (67/33%)	0.86
GCS M, median (IQR)	5 (5–6)	6 (5–6)	5 (4–6)	***0.001***
Pupillary abnormality, *n* (%)	13 (5%)	0 (0%)	13 (7%)	***0.03***
WFNS grade (IV–V/I–III), *n* (%)	183/59 (76/24%)	39/24 (62/38%)	144/35 (80/20%)	***0.003***
Hunt and Hess grade (IV–V/I–III), *n* (%)	110/132 (55/45%)	18/45 (29/71%)	92/87 (51/49%)	***0.002***
Fisher grade, median (IQR)	4 (3–4)	3 (3–4)	4 (3–4)	***0.002***
Aneurysm, anterior/posterior circulation, *n* (%)	196/46 (81/19%)	47/16 (75/25%)	149/30 (83/17%)	0.13
Aneurysm treatment, no/endovascular/surgery/both, *n* (%)	4/169/67/2 (2/70/28/1%)	0/50/11/2 (0/79/18/3%)	4/119/56/0 (2/66/31/0%)	***0.01***
Thiopental, *n* (%)	24 (10%)	2 (3%)	22 (12%)	***0.04***
Decompressive craniectomy, *n* (%)	34 (14%)	5 (8%)	29 (16%)	0.10

*p* Values in bold italics are considered statistically significant

Of the 242 patients, 63 (26%) had favorable and 179 (74%) unfavorable clinical outcome (Table 1). Those with favorable outcome were significantly younger, in a better neurological condition at admission, had less SAH on the first CT scan and were more often treated with endovascular aneurysm occlusion.

### Temporal Dynamics of Systemic and Cerebral Physiological Variables, and Relation to Triple-H Treatment and Clinical Outcome

**Fig. 1:**
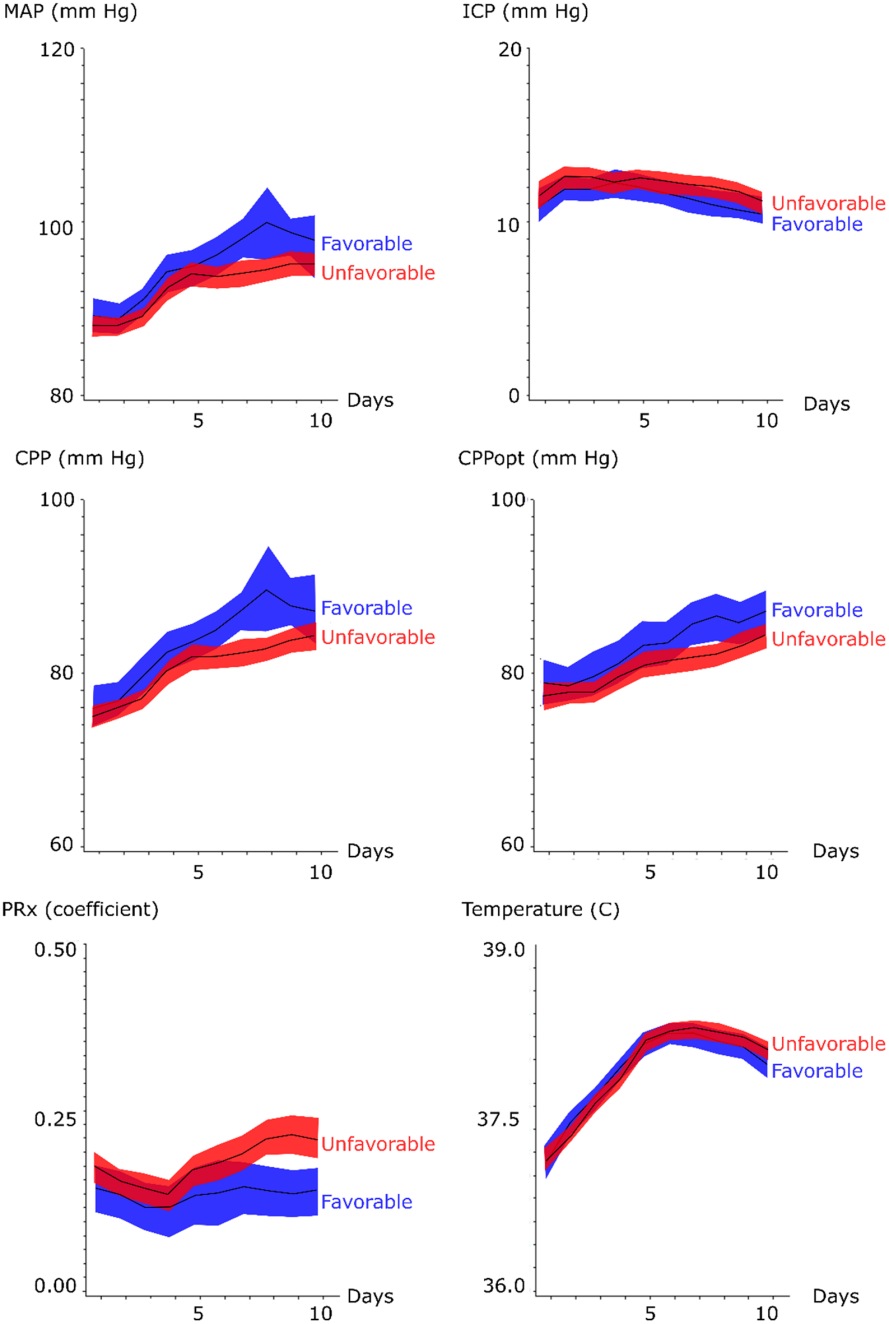
Systemic and cerebral physiological variables in relation to clinical outcome following aneurysmal subarachnoid hemorrhage—a temporal analysis the first 10 days. The trendlines indicate mean values and the shaded areas (red and blue) the 95% confidence interval. CPP = Cerebral perfusion pressure, CPPopt = optimal CPP (the CPP with the concurrently best autoregulation), ICP = Intracranial pressure, MAP = Mean arterial blood pressure, and PRx = Pressure reactivity index

**Fig. 2:**
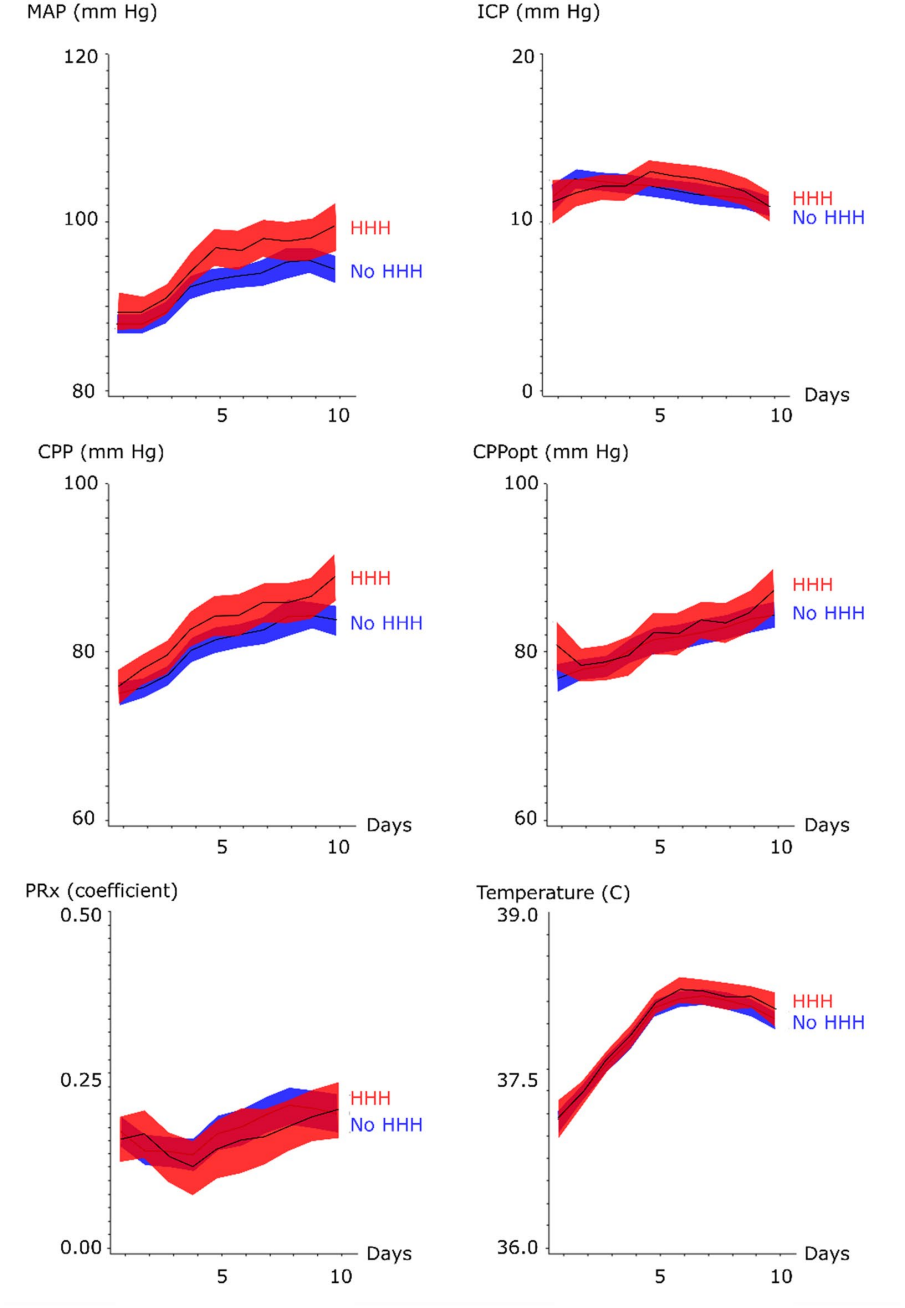
Systemic and cerebral physiological variables in relation to triple-H therapy following aneurysmal subarachnoid hemorrhage—a temporal analysis the first 10 days. The trendlines indicate mean values and the shaded areas (red and blue) the 95% confidence interval. CPP = Cerebral perfusion pressure, CPPopt = optimal CPP (the CPP with the concurrently best autoregulation), ICP = Intracranial pressure, MAP = Mean arterial blood pressure, and PRx = Pressure reactivity index

Mean daily values of systemic and cerebral physiological variables the first 10 days post-hemorrhage for all patients are demonstrated in Figs. 1 and 2. The mean daily MAP at admission was around 90 mm Hg, gradually increased throughout the temporal course to 100 mm Hg, and was significantly higher for those with favorable outcome in the late vasospasm phase. Those who were treated with triple-H had higher MAP in the early and late vasospasm phases. Mean ICP remained relatively stable around 10 mm Hg throughout the temporal course for both outcome groups. The temporal CPP trend was similar to the MAP curve, with a mean daily CPP that was slightly lower at admission (below 80 mm Hg), gradually increased along the temporal course and was significantly higher for those with favorable outcome in the early and late vasospasm phases. Similarly, those treated with triple-H had higher CPP in the same phases. The CPPopt trend showed a similar pattern as for mean CPP and CPPopt was significantly higher in the early (83 ± 10 mm Hg vs. 81 ± 8 mm Hg, *p *value < 0.05) and late (87 ± 9 mm Hg vs. 83 ± 8 mm Hg, *p *value < 0.01) vasospasm phases for those with favorable outcome. PRx was generally disturbed with mean values above 0 throughout the temporal course for both outcome groups. Those with unfavorable outcome had significantly higher PRx in the late vasospasm period than those with favorable outcome (0.22 ± 0.16 vs. 0.14 ± 0.12, *p *value < 0.001). Those with triple-H treatment did not have a significantly higher PRx. Body temperature increased in both outcome groups from approximately 37 °C at admission to slightly above 38 °C in the early and late vasospasm phases.

### Temporal Occurrence of Predefined Secondary Insults and Relation to Clinical Outcome

**Fig. 3:**
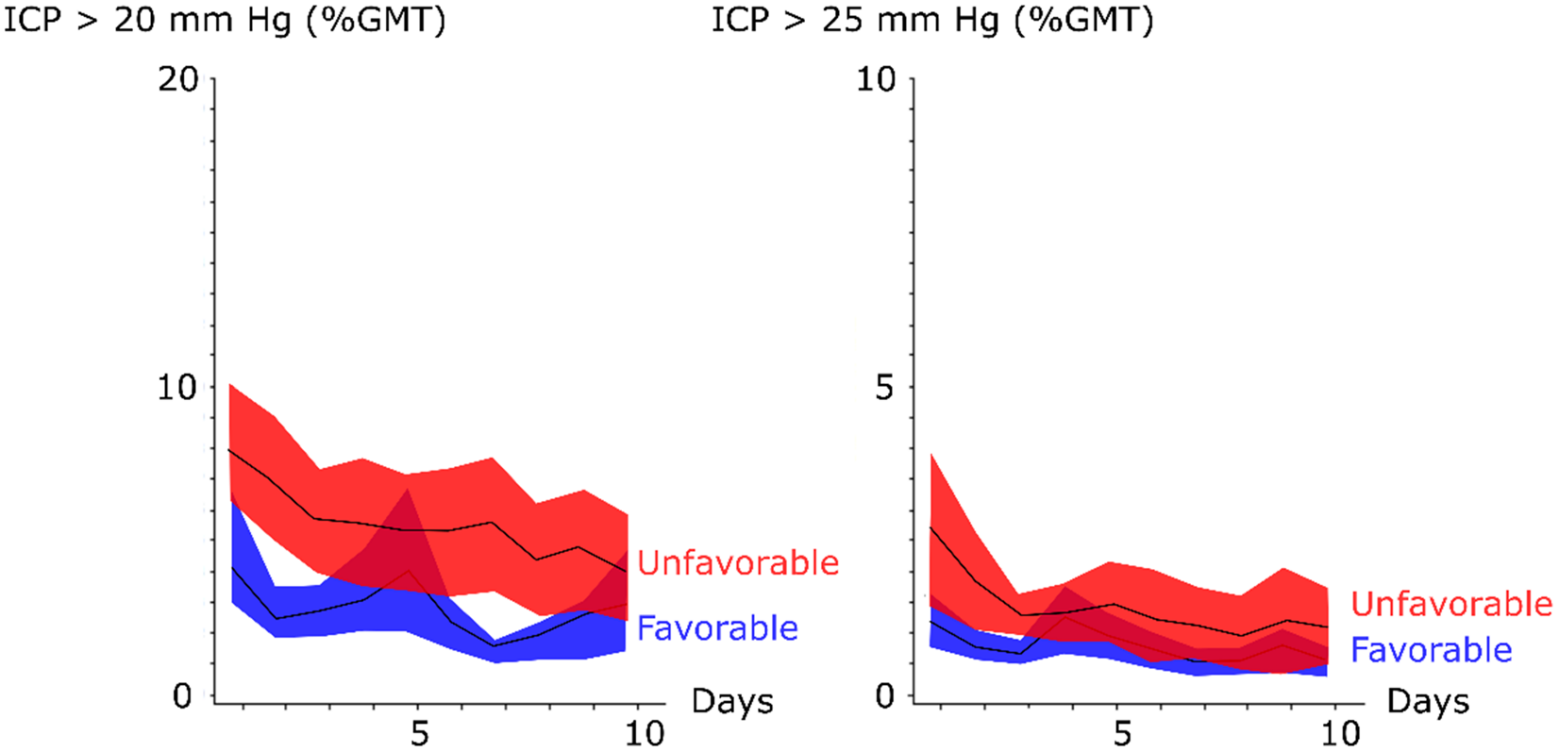
Intracranial pressure thresholds in relation to clinical outcome following aneurysmal subarachnoid hemorrhage—a temporal analysis the first 10 days. The trendlines indicate mean values and the shaded areas (red and blue) the 95% confidence interval. GMT = Good monitoring time and ICP = Intracranial pressure

The incidence of ICP insults (GMT (%) ICP > 20 mm Hg and GMT (%) > 25 mm Hg, respectively) were higher in the early phase post-ictus (Fig. 3 and Table 2). Patient with favorable outcome had significantly lower GMT (%) of ICP above both thresholds in the early phase (above 20 mm Hg, 3 ± 3 vs. 6 ± 9, *p *value < 0.001 and above 25 mm Hg 1 ± 1 vs. 2 ± 2, *p *value < 0.001) (Table 2). Those with favorable outcome also had significantly lower GMT (%) of ICP insults above 20 mm Hg in the early and late vasospasm phases (Table 2).

**Table 2:** Secondary insults in relation to clinical outcome following aneurysmal subarachnoid hemorrhage—a temporal analysis of three phases the first 10 days

Variables	Ictal phase			Early vasospasm phase			Late vasospasm phase		
	Favorable	Unfavorable	*p *Value	Favorable	Unfavorable	*p *Value	Favorable	Unfavorable	*p *Value
ICP > 20 mm Hg (%)	3 (± 3)	6 (± 9)	***0.001***	3 (± 4)	5 (± 13)	***0.04***	2 (± 3)	4 (± 12)	***0.01***
ICP > 25 mm Hg (%)	1 (± 1)	2 (± 2)	***0.001***	1 (± 1)	1 (± 3)	0.30	1 (± 1)	1 (± 4)	0.35
CPP < 60 mm Hg (%)	4 (± 5)	6 (± 7)	0.09	2 (± 3)	3 (± 4)	***0.01***	1 (± 2)	2 (± 5)	***0.02***
CPP < 70 mm Hg (%)	26 (± 20)	32 (± 19)	0.05	14 (± 14)	19 (± 17)	0.05	8 (± 9)	15 (± 17)	***0.001***
CPP < 80 mm Hg (%)	59 (± 24)	66 (± 21)	**0.04**	41 (± 27)	48 (± 26)	0.06	27 (± 21)	40 (± 27)	***0.001***
CPP < 90 mm Hg (%)	84 (± 15)	87 (± 14)	0.08	69 (± 27)	75 (± 23)	0.09	55 (± 27)	69 (± 25)	***0.001***
∆CPPopt < -10 mm Hg (%)	25 (± 14)	25 (± 13)	0.94	18 (± 12)	20 (± 12)	0.44	18 (± 11)	20 (± 11)	0.19
∆CPPopt ± 10 mm Hg (%)	55 (± 12)	56 (± 11)	0.67	56 (± 11)	54 (± 10)	0.20	53 (± 12)	54 (± 11)	0.43
∆CPPopt > 10 mm Hg (%)	16 (± 9)	15 (± 9)	0.42	21 (± 12)	21 (± 13)	0.66	25 (± 15)	21 (± 12)	0.08
PRx (coefficient)	0.13 (± 0.12)	0.15 (± 0.13)	0.18	0.14 (± 0.16)	0.17 (± 0.15)	0.18	0.15 (± 0.12)	0.22 (± 0.16)	***0.001***
PRx > 0.05 (%)	59 (± 15)	61 (± 15)	0.42	60 (± 19)	62 (± 16)	0.38	61 (± 14)	67 (± 17)	***0.01***
Body temperature > 38C (%)	15 (± 19)	13 (± 16)	0.44	55 (± 29)	54 (± 27)	0.93	51 (± 29)	61 (± 28)	***0.01***

*p* Values in bold and bold italics are considered statistically significant

**Fig. 4:**
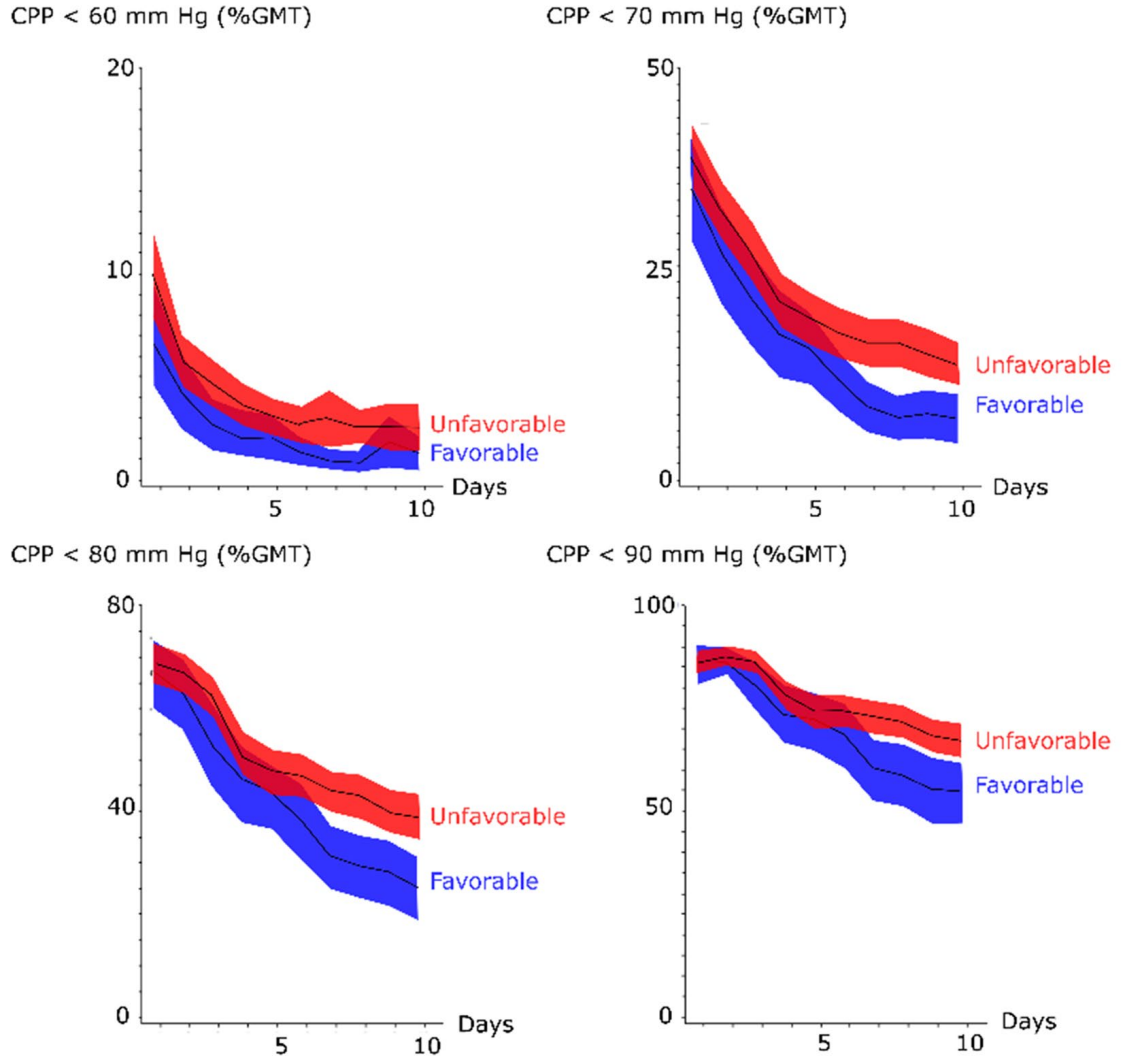
Fixed cerebral perfusion pressure thresholds in relation to clinical outcome following aneurysmal subarachnoid hemorrhage—a temporal analysis the first 10 days. The trendlines indicate mean values and the shaded areas (red and blue) the 95% confidence interval. CPP = Cerebral perfusion pressure and GMT = Good monitoring time

**Fig. 5:**
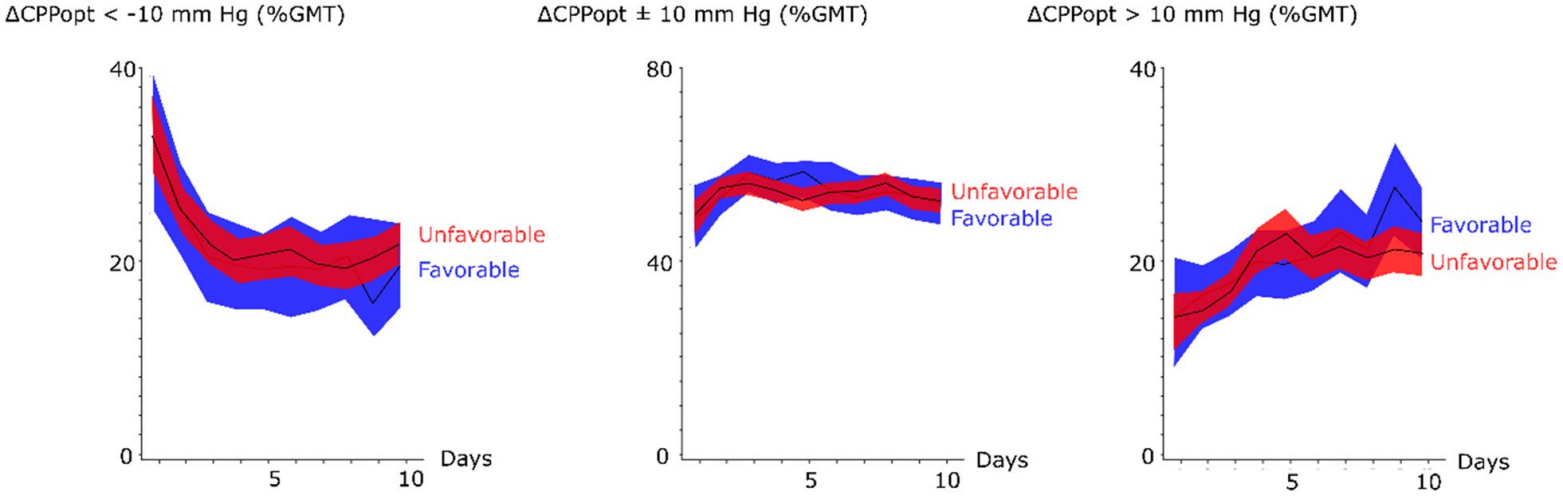
Autoregulatory cerebral perfusion pressure thresholds in relation to clinical outcome following aneurysmal subarachnoid hemorrhage—a temporal analysis the first 10 days. The trendlines indicate mean values and the shaded areas (red and blue) the 95% confidence interval. CPP = Cerebral perfusion pressure, CPPopt = optimal CPP (the CPP with the concurrently best autoregulation), ∆CPPopt = CPP–CPPopt, and GMT = Good monitoring time

The incidence of CPP below 60, 70, 80, and 90 mm Hg was higher in the early phase post-ictus and gradually decreased (Fig. 4 and Table 2). Patients with favorable outcome had significantly lower GMT (%) of CPP < 80 mm Hg (59 ± 24 vs. 66 ± 21, *p *value < 0.05), but the other fixed CPP thresholds did not differ in the early phase (Fig. 4 and Table 2). There was no association between favorable outcome and GMT (%) of the fixed CPP thresholds in the early vasospasm phase, but favorable outcome was very significantly associated with lower GMT (%) below all CPP thresholds (from 60 to 90 mm Hg) in the late vasospasm phase (Table 2). There was no association between clinical outcome and ∆CPPopt thresholds in any phase (Fig. 5 and Table 2) and this held true even after excluding those patients with DC surgery (data not presented).

Both outcome groups had a mean PRx above 0 in the early phase and both vasospasm phases (Fig. 1 and Table 2) and it was only significantly lower for those with favorable outcome in the late vasospasm phase (0.15 ± 0.12 vs. 0.22 ± 0.16, *p *value < 0.001). Exclusion of those who were operated with DC did not affect this association (data not presented). Both outcome groups also had similar incidence of systemic hyperthermia insults in the first two phases and those with favorable outcome had significantly lower incidence in the late vasospasm phase (51 ± 29 vs. 61 ± 28, *p *value < 0.01) (Table 2).

### Multiple Logistic Regression of Favorable Outcome for the Three Phases

Multiple logistic regression for favorable outcome was performed for each of the three phases including the secondary insult variables that were significant in the univariate analyses in addition to age, WFNS grade, and Fisher grade as explanatory variables (Table 3). Younger age, lower WFNS grade, and lower Fisher grade were significantly associated with favorable outcome in all three phases. In the early phase, lower GMT (%) with ICP above 20 mm Hg was also significantly associated with favorable outcome (AUROC = 0.793), as was lower GMT (%) with ICP above 25 mm Hg (AUROC = 0.792) in a separate outcome regression (not shown). In the early vasospasm phase, the incidence of secondary ICP and CPP insults were not independent predictors of favorable outcome, but a lower incidence of CPP below 90 mm Hg in the late phase was associated with favorable outcome. The other CPP thresholds in the same phase were not independent predictors when analyzed separately. Lower mean PRx in the late phase was independently associated with favorable clinical outcome (AUROC = 0.807), as was lower GMT (%) PRx > 0.05 (AUROC = 0.803) in a separate regression (not shown). However, ICP > 20 mm Hg and body temperature > 38 °C were not independent outcome predictors in that phase.

**Table 3:** The relation between secondary insults and clinical outcome in three phases following aneurysmal subarachnoid hemorrhage—multiple logistic regression of favorable outcome

Variables	Ictal phase	
	Odds ratio (95%CI)	*p *Value
Age	0.92 (0.89–0.95)	***0.001***
WFNS grade	0.77 (0.59–0.99)	***0.05***
Fisher grade	0.54 (0.31–0.95)	***0.03***
ICP > 20 mm Hg	0.83 (0.74–0.93)	***0.001***
CPP < 80 mm Hg	1.0 (0.98–1.0)	0.49

Variables	Early vasospasm phase	*p *Value
	Odds ratio (95%CI)	
Age	0.94 (0.91–0.97)	***0.001***
WFNS grade	0.71 (0.55–0.91)	***0.01***
Fisher grade	0.39 (0.22–0.70)	***0.002***
ICP > 20 mm Hg	0.95 (0.89–1.01)	0.07
CPP < 60 mm Hg	0.96 (0.87–1.07)	0.49

Variables	Late vasospasm phase	*p *Value
	Odds ratio (95%CI)	
Age	0.94 (0.91–0.97)	***0.001***
WFNS grade	0.72 (0.53–0.96)	***0.03***
Fisher grade	0.37 (0.19–0.72)	***0.003***
ICP > 20 mm Hg	0.92 (0.82–1.04)	0.17
CPP < 90 mm Hg	0.99 (0.97–0.99)	***0.04***
*T* > 38 °C	0.99 (0.98–1.00)	0.19
PR_*x*_	0.02 (0.00–0.25)	***0.003***

*p* Values in bold italics are considered statistically significant

All three outcome regressions included age, WFNS grade, and Fisher grade as baseline variables. The secondary insult variables that were significant in the univariate analyses in each phase were analyzed. When several thresholds of, e.g., ICP (20 and 25 mm Hg) in the early phase were associated with clinical outcome, both thresholds were analyzed in separate regressions and the regression with the highest area under receiver operating curve was presented in the current table

## Discussion

In the current study of 242 patients with severe aSAH that required ICP monitoring, the incidence of secondary insults and their relation to clinical outcome were related to different temporal phases post-ictus. In the early phase, ICP insults above 20 mm Hg were more frequent and independently associated with unfavorable outcome. In the late vasospasm phase, lower CPP levels (even regarding as high thresholds as 80–90 mm Hg) were associated with unfavorable outcome. Pressure autoregulation was disturbed in the majority of patients throughout the temporal course and most evidently so in the late vasospasm phase for those with unfavorable outcome. Deviations of absolute CPP from the PRx-derived CPPopt target were not associated with unfavorable outcome. Our findings indicate that more aggressive ICP treatment in the early phase and that higher CPP above our traditional threshold at 60 mm Hg in the vasospasm phase could be beneficial.

### Intracranial Pressure in Aneurysmal Subarachnoid Hemorrhage–Thresholds and Timing of Insults

ICP rises rapidly following intracranial aneurysm rupture and may cause an intracranial circulatory arrest. ICP may remain elevated in the early course due to global cerebral edema, acute hydrocephalus, and intracranial hemorrhage volume and in the later phase following complications to aneurysm treatment and DCI. Several previous studies have reported a relatively high incidence of ICP above 20 or 25 mm Hg [4, 7–9], but the relation between high ICP and clinical outcome is not fully elucidated. We have previously reported that higher GMT (%) of ICP above 25 mm Hg was associated with clinical deterioration in aSAH, but not with long-term clinical outcome [4]. Others have reported an association between ICP above 20 mm Hg and mortality [7], but not as an independent predictor of unfavorable clinical outcome [7, 8]. In the current study, we evaluated the temporal dynamics of ICP insults and found that they were more frequent in the early phase post-ictus and independently predicted unfavorable outcome. This association to clinical outcome could be confounded by the detrimental etiologies for increased ICP, as mentioned above, but it held true even after adjustment for the neurological status at admission (WFNS) and structural injury (Fisher grade). This finding suggests that ICP above 20 mm Hg is dangerous and should be avoided.

Regarding specific ICP thresholds, we found that higher GMT (%) of both ICP above 20 mm Hg and 25 mm Hg, respectively, was strong predictor of unfavorable outcome. This supports 20 mm Hg as a reasonable ICP threshold after aSAH, consistent with the management protocol at our NIC and many other sites [7, 8]. It also supports that similar ICP thresholds can be used in both aSAH and TBI [27]. However, it is possible that although ICP above 20 mm Hg might dichotomize between acceptable and dangerously elevated ICP, lower ICP targets might be beneficial to increase CPP during vasospasm/DCI.

### Pressure Autoregulation and Cerebral Perfusion Pressure in Aneurysmal Subarachnoid Hemorrhage—Fixed and Autoregulatory Thresholds

Cerebral pressure autoregulation is commonly disturbed after aSAH with development of large-vessel cerebral vasospasm that peaks between days 4 to 10 after ictus [5]. Several studies have used TCD-derived measures of vasospasm, but this method is user-dependent and does not allow for continuous measurements [11]. PRx is a well-established method for continuous evaluation of pressure autoregulation in TBI [12–14], however, its role in aSAH is less established. Previous studies on PRx in aSAH demonstrate that higher values indicative of disturbed pressure autoregulation are common and associated with lower CBF [16], but its relation to the risk of developing DCI is controversial [16, 22]. The association between PRx and clinical outcome is also not clear. Gaasch et al. found that PRx was significantly higher in the early phase post-ictus for patients that later developed DCI and had unfavorable outcome [22], but others have found no association between PRx and clinical outcome [20, 21]. However, the earlier publications are based on small patient populations (< 100) and this is to our knowledge the largest study on PRx in aSAH including 242 patients. We found that PRx was commonly above zero throughout the temporal course, indicating disturbed pressure autoregulation. PRx was also significantly higher in the late vasospasm phase for those with unfavorable outcome, but not earlier and not worse for those who developed DCI and required triple-H therapy. It is possible that the deterioration in PRx during the late vasospasm phase reflects a larger burden of silent DCI in these patients that explains a worse clinical outcome. However, as PRx varies with CPP [15], it could also be confounded by that patients with favorable outcome also had higher CPP.

In addition to being a risk factor for poor outcome and possibly DCI, PRx can be used for estimation of optimal CPP (CPPopt), i.e., the CPP that gives the lowest PRx, that can be used to individualize CPP targets [13–15, 18, 19]. Several retrospective studies in TBI have indicated that keeping the absolute CPP close to the CPPopt target could improve clinical outcome [13–15] and CPPopt management is currently being evaluated in a randomized controlled trial (RCT) in TBI (COGiTATE) [28]. Deviation from CPPopt has also been associated with worse outcome in aSAH in a smaller study including 29 patients [19], but it has not been validated in larger patient cohorts or compared with fixed CPP targets.

In the current study, CPPopt was higher for those with favorable outcome. This is paradoxical, since higher CPPopt would indicate a need to counteract an increased cerebrovascular tone such as in cases with cerebral vasospasm, whereas vasospasm is usually associated with worse clinical outcome. Future studies are needed to further explore this somehow surprising association. However, we found that there was no association between deviations of absolute CPP from CPPopt and clinical outcome in any phase post-ictus. Nevertheless, we did find that higher absolute CPP was generally associated with more favorable outcome, specifically in the vasospasm phase. The etiology for higher CPP in the vasospasm phase was rather explained by higher MAP than lower ICP (Fig. 1). The lower CPP threshold in our management protocol is 60 mm Hg, but our results suggest that even higher values above 90 mm Hg could be beneficial in the late vasospasm phase. These findings are consistent with Schmidt et al., who found that the risk of detrimental brain tissue hypoxia and energy metabolic disturbances is relatively high even when CPP is between 60 and 70 mm Hg, but decreases above 70 mm Hg [10].

Our results highlight that although several management principles including CPP thresholds are extrapolated from severe TBI to aSAH, the relation between CPP and clinical outcome differs. In TBI, we have previously found that CPP between 60 and 70 mm Hg is associated with favorable outcome and CPP above 70 mm Hg is associated with worse clinical outcome [13, 29], whereas we found that higher CPP above 70 mm Hg was better in our aSAH patients in the current study. CPPopt targets have shown promising results in TBI [13–15], but not in the current study on aSAH. Fixed CPP targets that are slightly increased during the vasospasm phase might suit the majority of aSAH patients with a relatively homogenous pathophysiology, whereas individualized autoregulatory CPPopt targets might be more appropriate in TBI victims with a more heterogeneous pathophysiology. The lack of correlation between CPPopt targets and clinical outcome in aSAH patients could also be explained by that PRx and CPPopt might have a lower signal-to-noise ratio in aSAH patients that generally are treated with open EVDs. However, we and others have demonstrated that the validity of PRx and hence CPPopt is preserved when the EVD is opened [30, 31]. Another explanation is that PRx is a global measure of pressure autoregulation, but vasospasm and DCI may be focal events [32]. Focal differences in the cerebrovascular function could even each other out when evaluated with a global measure, which could reduce the sensitivity of such events for PRx.

Although our findings indicate that higher CPP is better in the vasospasm phase, this does not necessarily support the use of vasopressors, since our patients were treated with vasopressors only if CPP was below 60 mm Hg, but not to higher levels. The findings may, however, at least indicate that spontaneously high blood pressure levels (CPP levels) within reasonable limits should not be lowered. We did not analyze the explanatory variables for differences in absolute CPP levels, but is likely that patients with comorbidities, aSAH-induced cardiac dysfunction, and systemic vasodilation from hyperthermia generally had lower MAP and CPP. Future prospective trials are needed to address if higher CPP targets in the vasospasm phase could improve clinical outcome after aSAH and what treatments that should be initiated to increase CPP, e.g., including more aggressive ICP reduction with lower EVD opening pressure and MAP-augmentation by fluids and vasopressors. It is also likely that optimal MAP management depends on the concurrent condition as mentioned above.

### Methodological Strengths and Limitations

The study included a uniquely, large patient population with high-resolution physiological data. We used a strict inclusion criteria so that all patients had ICP/CPP data on all of the first 10 days, for better evaluation of the temporal dynamics in physiological variables. Hence, our patient population mainly reflects those with the most severe aSAH that required such extensive ICP monitoring, since the EVD may not be needed or can usually be discontinued earlier in more benign cases. This limits the external validity of our findings to those with the most severe aSAH. This also explains why the clinical outcome was generally poor in our study population (only 26% with favorable outcome). Furthermore, this was a retrospective, observational study and it is likely that many confounding variables could explain the relation between physiological variables and clinical outcome. We addressed this to some extent by multiple regression analyses, but we cannot exclude that other unadjusted variables still confounded the results. There are also some concerns about the validity of PRx and CPPopt after DC. Recent findings in TBI suggest that these measures are still valid after DC [33] and we found no impact on the results including PRx and CPPopt after excluding the patients with DC surgery. In addition, CPPopt might be relatively high in case of cerebral vasospasm and we cannot exclude that we did not reach high enough CPP values that fully explored the upper limit of the CPPopt curve. It is possible that a more aggressive CPP management toward higher values would have better revealed a truer CPPopt and that keeping the absolute CPP close to such values would have better discriminated between favorable and unfavorable outcome. It is also possible that the absolute PRx values were too high in general for CPPopt curves to occur.

## Conclusions

The incidence of secondary insults including intracranial pressure above 20 mm Hg and cerebral perfusion insults below 60–70 mm Hg was higher for patients with unfavorable clinical outcome after aneurysmal subarachnoid hemorrhage. The former were particularly common in the early phase. The latter were less common in the vasospasm phase, but still correlated more strongly with worse clinical outcome then when the pressure autoregulatory status was worse. Our findings indicate that more aggressive intracranial pressure treatment in the early phase and a cerebral perfusion pressure target above 70 mm Hg in the vasospasm phase could be beneficial. Particularly, this suggests that antihypertensive agents should generally be avoided in the vasospasm phase, but it is possible that more aggressive intracranial pressure lowering treatments and/or the use of certain vasopressors could be favorable. There was no relation between deviations from CPPopt and clinical outcome, although we cannot exclude an association discernible in a larger patient population and with a different CPP management.
